# A systematic review of the spinal instability neoplastic score and its relationship with peri-interventional patient-centered measures: The clinical impact of spinal instability

**DOI:** 10.1097/MD.0000000000044992

**Published:** 2025-10-24

**Authors:** Hassan Darabi, Harshit Arora, Henry Ruiz-Garcia, Aysha Alsahlawi, Jared T. Wilcox, Francis Farhadi

**Affiliations:** a Department of Neurosurgery, College of Medicine, University of Kentucky, Lexington, KY.

**Keywords:** functional status, pain outcomes, patient-reported outcomes, quality of life, spinal instability, spinal metastasis

## Abstract

**Background:**

The spinal instability neoplastic score (SINS) provides a standardized assessment of spinal stability in patients with metastatic spine disease. Although intended to assist clinical decision-making, the relationships between SINS and patient-centered measures, such as pain intensity, functional status, and health-related quality of life (HRQoL), remain undefined.

**Methods:**

This systematic review followed PRISMA guidelines. A comprehensive literature search was performed across PubMed, Scopus, Embase, and Web of Science databases using keywords related to SINS and spinal metastases. Studies assessing the relationships between SINS and pain intensity scores, functional status, and HRQoL were included. Data on study characteristics, type of interventions, and patient-centered measures were extracted. Risk of bias was assessed using the Newcastle-Ottawa Scale. A meta-analysis was not feasible due to significant treatment, outcome, and population heterogeneity.

**Results:**

Thirteen studies (n = 1823; mean age 63.0 ± 12.5 years) were included. Five of six studies reported a significant association between higher baseline SINS scores and pain intensity, most commonly using the Visual Analog Scale and Numeric Rating, while 1 of 2 studies identified a predictive value of SINS for posttreatment pain. Nine studies evaluated peri-interventional functional status using 6 different tools; significant correlations with baseline SINS were identified for MD Anderson Symptom Inventory and Spine Oncology Study Group Outcomes Questionnaire 2.0, while no relationships were identified for the Barthel Index, Eastern Cooperative Oncology Group score, or Frankel scale. Further, stable postradiotherapy SINS was associated a higher baseline Karnofsky Performance Status (KPS). Three studies assessed HRQoL using either the European Organisation for Research and Treatment of Cancer Quality of Life Questionnaire-Core 30, the 36-Item Short Form Survey, or the EuroQol Five-Dimension Scale; 2 of these studies reported that higher SINS values were associated with lower baseline physical functioning. Of 13 studies, 12 were of moderate methodological quality.

**Conclusions:**

SINS demonstrated correlations with peri-interventional pain intensity, functional status, and HRQoL. Pretreatment correlations were generally more consistent. However, in radiotherapy-treated cohorts, stable posttreatment SINS was associated with higher baseline KPS, suggesting a potential predictive relationship between these measures.

## 1. Introduction

Metastatic spine disease is a debilitating condition that is becoming more common due to improvements in cancer care. In this context, accurate assessment of spinal stability is critical for guiding treatment decisions and to minimize negative impacts on quality of life and functional capacity.^[1–3]^ The spinal instability neoplastic score (SINS) provides a standardized method for evaluating the structural integrity of the spine in patients with metastatic lesions. SINS integrates both radiological and clinical parameters including tumor location, type of bony lesion, spinal alignment, vertebral body collapse, and pain characteristics to classify spinal stability into 3 categories: 0 to 6 indicates a stable spine, 7 to 12 reflects indeterminate or potential instability, and 13 to 18 denotes instability.^[4]^ By identifying individuals at higher risk of mechanical instability, the score can assist clinicians in selecting the most appropriate intervention, whether surgical, radiotherapeutic, or a combination of modalities.^[5]^

Numerous studies have demonstrated the reliability of SINS and its utility in clinical decision-making, including in predicting the need for surgical fixation and the incidence of vertebral compression fractures.^[6–8]^ While the association between SINS and survival has been a key focus of various studies,^[9–11]^ overall survival per se is not the sole consideration in this population. Given that palliative care needs are of primary importance, patient-centered measures, such as pain intensity, functional status, and health-related quality of life (HRQoL), represent important considerations.

Despite the growing adoption of SINS in routine practice,^[12,13]^ there remains a need for a clearer understanding of its relationship with peri-interventional patient-centered measures. This systematic review evaluates the influence of SINS on these measures. By elucidating this relationship, we aim to assess the clinical relevance of SINS beyond its traditional role in guiding surgical treatment and to explore its potential utility as a baseline marker that can potentially correlate with peri-interventional patient-centered measures.

## 2. Methods

This systematic review was conducted in line with preferred reporting items for systematic reviews and meta-analyses (PRISMA). No ethical approval was required for this study.

### 2.1. Search strategy

Using a comprehensive search strategy focused on keywords related to SINS scoring, 2 independent reviewers conducted searches across 4 major databases: PubMed, Scopus, Embase, and Web of Science. The initial search was completed in January 2025. The search strategy incorporated various keyword variations of “SINS” and, where applicable, included controlled vocabulary such as Medical Subject Headings (MeSH) in PubMed and Emtree terms in Embase. Additionally, the reviewers examined gray literature sources, including conference proceedings and published abstracts. The full search strategy is as follows:

(“spinal instability neoplastic score” OR “SINS” OR “SINS score”) AND (“Spine”[Mesh] OR “SPIN*”) AND (“Neoplasm Metastasis”[Mesh] OR “metasta*”)

### 2.2. Study selection

Two independent reviewers (HD, HA) conducted a systematic screening process, beginning with a title and abstract review, followed by a full-text assessment based on predefined inclusion and exclusion criteria. The inclusion criteria covered all human studies that utilized SINS (Table 1) to evaluate patient-centered measures including pain intensity, functional status and HRQoL metrics in individuals with spinal metastases, either before or after surgery. Studies involving nonhuman subjects, cadaveric models, reviews, technical notes, editorials, and correspondence letters were excluded. Additionally, studies that reported outcomes exclusively within a single SINS category, such as only potentially unstable patients, were also excluded. Any potential discrepancies regarding article eligibility were resolved through discussion with a third reviewer (FF).

**Table 1:** Spinal Instability Neoplastic Score

Domain	Points
Location of metastasis	3 = junctional zones (occiput–C2; C7–T2; T11–L1; L5–S1) 2 = mobile segments (C3–C6; L2–L4) 1 = semi-rigid thoracic (T3–T10) 0 = rigid distal sacrum (S2–S5)
Mechanical pain	3 = pain worsened by motion/axial loading and eased by recumbency 1 = occasional, nonmechanical pain 0 = no pain
Lesion type	2 = lytic 1 = mixed lytic/blastic 0 = blastic
Alignment	4 = vertebral translation or frank subluxation 2 = new kyphotic/scoliotic deformity 0 = normal alignment
Vertebral body integrity	3 = >50% body height loss 2 = <50% body height loss 1 = no collapse but >50% body involvement 0 = none of the above
Posterolateral structure involvement	3 = bilateral 1 = unilateral 0 = none

### 2.3. Data extraction

Baseline study characteristics, such as author name, year of publication, country of origin, study design, type of intervention, sample size, patient sex, age, follow-up interval, metastasis origin and type of patient-centered measures evaluated were extracted by reviewers.

### 2.4. Risk of bias assessment

Two independent reviewers assessed the risk of bias in the included studies using the Newcastle-Ottawa Scale,^[14]^ which has a maximum score of 9 points. Based on their scores, the studies were classified into 3 categories: low quality (0–3 points), moderate quality (4–6 points), and high quality (7–9 points).

### 2.5. Statistical analysis

The study findings were summarized using descriptive statistics, including frequencies, percentages, means, and standard deviations. A meta-analysis was not feasible due to significant heterogeneity with respect to the manner of patient-centered outcome measure reporting. Notably, all percentage values presented in the results were derived based on the total number of patients reported in the respective studies for each outcome.

## 3. Results

### 3.1. Overview of the included studies

**Figure 1.**
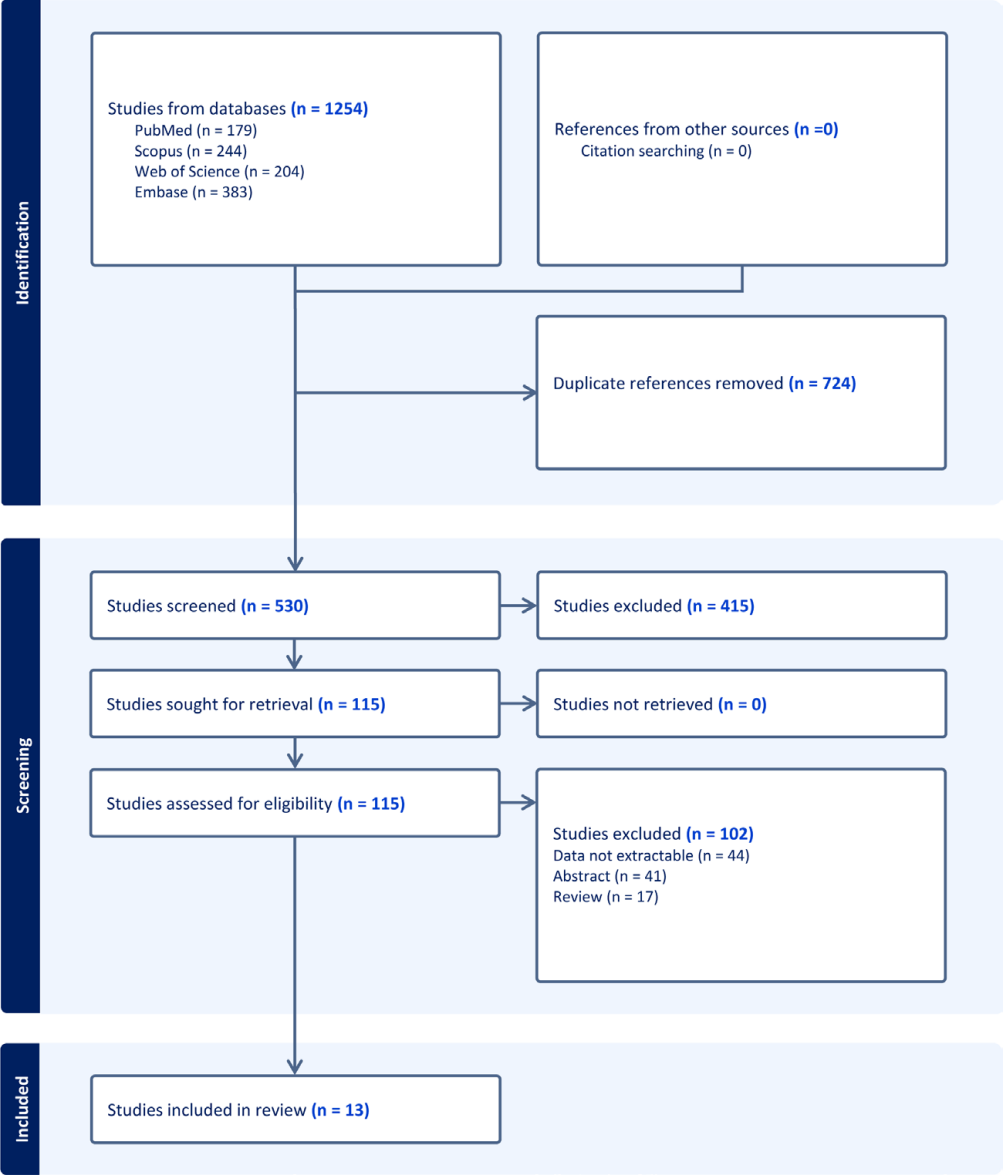
PRISMA flow diagram for study selection.

The initial literature search across the databases yielded a total of 1254 articles. After removing duplicates, 530 articles were selected for title and abstract screening, and the full text of 115 articles was reviewed. In total, 13 studies were included (Fig. 1).

### 3.2. Baseline study characteristics

A total of 1823 patients from 13 studies were included in this review. Sex distribution was reported for 1713 patients (947 males [55.3%] and 765 females [44.7%]), while 1 cohort of 110 patients did not specify sex.^[15–27]^ The overall mean age was 63 ± 12.5 years. The follow-up interval ranged from 0.1 to 128 months. The studies were conducted in Japan (3), Germany (2), Sweden (1), Canada (1), Brazil (1), Australia (1), Italy (1), the USA (1), and Korea (1). The study designs varied, with 8 retrospective and 5 prospective studies. Interventions included radiotherapy alone (5 studies), surgery alone (7 studies), and combined surgery with radiotherapy (1 study). The most common primary tumor origins were lung (reported in 8 studies), breast (reported in 8 studies), gastrointestinal (GI) (reported in 5 studies), prostate (reported in 5 studies) and kidney (reported in 4 studies). A total of 13 patient-centered measures were evaluated including: Visual Analog Scale (VAS), Numeric Rating Scale (NRS), Brief Pain Inventory (BPI), FACES Scale, Karnofsky Performance Status (KPS), the Spine Oncology Group Outcome Questionnaire 2.0 (SOSGOQ2.0), MD Anderson Symptom Inventory (MDASI), Barthel Index, Frankel scale, Eastern Cooperative Oncology Group (ECOG), European Organisation for Research and Treatment of Cancer Quality of Life Questionnaire (EORTC QLQ-C30), the 36-Item Short Form Survey (SF-36), and the EuroQol Five-Dimension Scale (EQ-5D) (Table 2).

**Table 2:** Baseline participant characteristics and outcome measures.

Authors (year)	Country	Study design	Intervention	Sample size (male: female)	Age, yrs (mean ± SD)	Follow-up interval	Most common metastasis origin	Outcomes
Cavalcante et al (2017)^[18]^	Brazil	Prospective	Surgery	*79* (41: 38)	57.9 ± 16.2	3 mo	Prostate (23), Breast (18), Lung (8)	VAS
Gallizia et al (2017)^[19]^	Italy	Prospective	RT	121 (67: 54)	62 ± 14	Median: 18 wk (range: 2–72 wk)	Breast (35), Lung (29), Prostate (22)	ECOG, VAS
Hussain et al (2018)^[20]^	USA	Prospective	Surgery	131 (75:56)	61.4 ± 12.5	Mean: 24.4 d (range: 16–125 d)	Lung (29), Kidney (18), Breast (12)	BPI, MDASI
Masuda et al (2018)^[22]^	Japan	Retrospective	Surgery	44 (30: 13)	66.5 ± 9.3	Mean: 28.3 ± 32.7 months (range: 1.0–128.0 mo)	Lung (13), Prostate (8), Breast (4)	ECOG, Frankel
Bostel et al (2020)^[16]^	Germany	Retrospective	RT	*66* (53: 13)	60.8 ± 14.4	Mean: 8.1 months (range: 0.3–85 mo)	HNC (57)	KPS
Donnellan et al (2020)^[26]^	Australia	Retrospective	Surgery	134 (79: 55)	61.3 ± 16	Variable (up to last follow-up or death)	Breast (22), Kidney (13), GI (12)	Modified Frankel index
Akezaki et al (2021)^[15]^	Japan	Retrospective	RT	79 (40:39)	65 ± 11.5	1 and 3 mo	Lung (25), Breast (19), GI (11)	EORT QLQ-C30
Bostel et al (2021)^[17]^	Germany	Retrospective	RT	*221* (97: 124)	63 ± 13.5	Median: 10.9 mo (range: 0.1–100.6 mo)	Visceral (84), GI (38), Lung (33)	KPS
Wänman et al (2021)^[27]^	Sweden	Retrospective	Surgery	110 (n/s)	71.4 ± 8.7	1 mo	Prostate (110)	Frankel index
Versteeg et al (2023)^[25]^	Canada	Prospective	Surgery ± RT	*307* (139: 168)	59.2 ± 10.2	6, 12, and 26 wk	Breast (84), Lung (52), Kidney (52)	SOSGOQ2, NRS, SF-36, EQ-5D
Kang et al (2024)^[21]^	Korea	Retrospective	Surgery	106 (61: 45)	58.2 ± 10.1	Variable (up to last follow-up or death)	Lung (28), GI (27), Breast (13)	ECOG, Frankel index, KPS
Nakata et al (2024)^[24]^	Japan	Retrospective	RT	108 (56: 52)	66 ± 15.2	1, 2, 3, 4, and 6 mo	Lung (39), GI (26), Breast (20)	NRS
Nakajima et al (2025)^[23]^	Japan	Prospective	Surgery	317 (209: 108)	66.7 ± 10.4	Median: 11 mo (range: 6–11 mo)	Lung (57), Kidney (46), Breast (39)	Barthel, EQ-5D, Faces Scale, VAS

Barthel = Barthel Index, BPI = Brief Pain Inventory, ECOG = Eastern Cooperative Oncology Group Performance Status, EORTC QLQ-C30 = European Organization for Research and Treatment of Cancer Quality of Life Questionnaire-Core 30, EQ-5D = EuroQol 5-Dimension questionnaire, FACES Scale = Faces Pain Scale, Frankel (or Modified Frankel) = Frankel grading system for spinal cord injury, GI = Gastrointestinal, HNC = head and neck cancer, KPS = Karnofsky Performance Status, MDASI = Metastatic Spinal Cord Compression-Modified Isolated/Functional Score, n/s = not specified, NRS = Numeric Rating Scale, RT = Radiotherapy, SD = standard deviation, SF-36 = 36-Item Short Form Survey, SOSGOQ2 = Spine Oncology Study Group Outcomes Questionnaire (version 2), VAS = visual analog scale.

### 3.3. SINS correlation with pain intensity

Six (46%) of the included studies investigated the relationship between SINS and pain intensity. They used 4 different pain assessment tools.^[18–20,23–25]^ The VAS was used in 3 studies^[18,19,23]^ while the NRS was reported in 2 studies.^[24,25]^ BPI and the FACES Scale were employed in a single study.^[20,23]^ Five articles identified a significant relationship between baseline pain and baseline SINS scoring system,^[18,20,23–25]^ while one of the articles did not find a statistically significant relationship.^[19]^ One of two studies identified a predictive value of SINS for postoperative pain.^[19]^ Among the various assessment tools used, the FACES Scale was the only one that showed no association with SINS^[23]^ (Table 3).

**Table 3:** Patient-centered measures and their relationship with the spinal instability neoplastic score (SINS).

Category	Variable	Description	Relationship with SINS
Pain assessment	VAS	A continuous 0–10 scale used to measure subjective pain intensity, with higher values indicating greater pain.	Higher SINS correlated with higher baseline VAS scores (*P* = .001) (Cavalcante et al, 2017^[18]^). SINS acted as a predictive factor of pain response after RT. A lower SINS corresponds to a higher possibility of pain control for both pain at rest (*P* = .007) and breakthrough pain (*P* = .047) (Gallizia et al 2017^[19]^). Higher SINS was significantly correlated with higher baseline VAS pain scores (*P* = .016); however, postoperative pain reduction did not differ significantly between SINS categories (stable, potentially unstable, or unstable) (Nakajima et al, 2025^[23]^).
	NRS	A discrete 0–10 scale where patients rate their pain intensity; 0 means no pain, and 10 is the worst imaginable pain.	Higher SINS was significantly correlated with higher pretreatment NRS pain scores (Versteeg et al, 2023^[25]^). Higher NRS is associated with higher baseline SINS**.** Regression analysis stratifying patients into low (≤4) and high (>4) NRS groups to predict factors for ongoing spinal instability at 1 month after RT did not reveal a significant correlation with SINS (*P* = .39) (Nakata et al, 2024^[24]^).
	BPI	Assesses both pain severity (worst, least, average, now) and pain interference with daily activities.	Higher SINS was significantly associated with greater preoperative pain severity indicated by BPI average and worst pain (*P* = .03). Also, walking was significantly limited due to pain interference (*P* = .04) Patients with SINS > 6 showed significant improvements in BPI after surgery (*P* < .05) (Hussain et al, 2018^[20]^).
	FACES scale	A visual scale using facial expressions to represent pain intensity.	None of the SINS categories (stable, potentially unstable, or unstable) correlated with different baseline or postoperative FACES scale (Nakajima et al, 2025^[23]^).
Functional status	Frankel score	A clinician-rated scale used to assess neurological function following spinal cord injury. It categorizes patients from Grade A (complete motor and sensory loss) to Grade E (normal neurologic function)	No significant difference between potentially unstable SINS (7–12) and unstable SINS (13–18) categories regarding postoperative Frankel index (Wänman et al, 2021^[27]^). No significant postoperative differences were noted in modified Frankel index across SINS groups as patients had favorable ambulatory function (Frankel grade D or E) after surgery, regardless of SINS classification (Donnellan et al, 2020^[26]^). No difference was observed between the SINS stable (SINS ≤ 12) and unstable (SINS > 12) groups, as both demonstrated significant postoperative improvements in the Frankel score (Masuda et al, 2018^[21]^).
	KPS	Rates patient functional ability on a scale from 0 (dead) to 100 (normal functioning).	Univariate modeling showed a KPS ≥ 70% was associated with 6-fold higher odds of stable SINS (SINS < 7) at 6 months after RT in patients with metastatic head and neck cancers (*P* = .006) (Bostel et al, 2020)^[16]^ and in patients with metastatic lung and breast cancer (*P* = .02) (Bostel et al, 2021^[17]^).
	ECOG	Ranges from 0 (fully active) to 5 (dead); assesses a patient’s ability to care for themselves and perform daily activities.	A higher SINS was not significantly correlated with ECOG scores either at baseline or following surgery (Kang et al, 2024^[21]^). No significant difference was observed between the stable (SINS ≤ 12) and unstable (SINS > 12) groups, as both demonstrated comparable improvements in the ECOG (Masuda et al, 2018^[22]^).
	Barthel index	Measures performance in basic activities of daily living, such as feeding, bathing, and walking; scores range from 0 (totally dependent) to 100 (independent).	None of the SINS categories (stable, potentially unstable, or unstable) correlated with different baseline or postoperative Barthel index (Nakajima et al, 2025^[23]^).
	SOSGOQ2.0	A spine-specific tool assessing physical function, pain, mental health, and neurologic symptoms in patients with spinal tumors.	Higher SINS was associated with worse SOSGOQ2 scores at baseline (*P* < .001) (Versteeg et al, 2023^[25]^).
	MDASI	A patient-reported scale assessing symptom severity and how symptoms interfere with daily activities in patients with spinal tumors.	Higher SINS was significantly correlated with MDASI pain (*P* = .03), activity (*P* = .006) and walking (*P* = .03) impairment preoperatively. Greater postoperative symptom relief was associated with higher SINS scores, as patients with elevated SINS experienced more significant reductions in MDASI pain (*P* = .04). (Hussain et al, 2018^[20]^).
HRQoL	EORTC QLQ-C30	A 30-item questionnaire measuring cancer-specific quality of life across physical, emotional, and social domains.	Classification in the unstable SINS group was significantly associated with higher global health status scores 1 month after radiotherapy (*P* < .05). In contrast, the physical function and emotional function domains of the EORTC QLQ-C30 did not demonstrate a significant relationship with SINS, with p-values of 0.12 and 0.09, respectively (Akezaki et al, 2021^[15]^).
	SF-36	A general health survey assessing 8 domains including physical functioning, pain, general health, vitality, and mental health.	Higher SINS correlated with lower baseline physical functioning on SF-36 (*P* < .001) (Versteeg et al, 2023^[25]^).
	EQ-5D	A standardized instrument measuring health-related quality of life across 5 dimensions (mobility, self-care, usual activities, pain/discomfort, anxiety/depression) and includes a visual analogue health scale.	Patients with higher SINS had lower EQ-5D scores at baseline. (*P* = .001) (Versteeg et al, 2023^[25]^). None of the SINS categories (stable, potentially unstable, or unstable) correlated with different baseline or postoperative EQ-5D (Nakajima et al, 2025^[23]^).

BPI = brief pain inventory, ECOG = Eastern Cooperative Oncology Group Performance Status, EORTC QLQ-C30 = European Organisation for Research and Treatment of Cancer Quality of Life Questionnaire, EQ-5D = EuroQol Five-Dimension Scale, FACES Scale = Wong-Baker Faces Pain Rating Scale, HRQoL = health-related quality of life, KPS = Karnofsky Performance Status, MDASI = MD Anderson Symptom Inventory, NRS = numeric rating scale for pain assessment, SF-36 = 36-Item Short Form Health Survey, SINS = spinal instability neoplastic score, SOGQ2 = Spine Oncology Group Questionnaire 2, VAS = visual analog scale for pain assessment.

### 3.4. SINS correlation with functional status

Nine (69%) of the included studies assessed the relationship between SINS and functional status, utilizing 6 different functional status tools.^[16,17,20–23,25–27]^ Frankel score^[22,26,27]^ was evaluated in 3 studies, while ECOG^[21,22]^ and KPS^[16,17]^ were each used in 2. All other measures, including MDASI,^[20]^ SOSGOQ2.0,^[25]^ and the Barthel Index,^[23]^ were assessed in only one study each. Three studies reported significant correlations between baseline SINS and posttreatment functional outcomes, namely KPS^[16,17]^ and MDASI.^[20]^ In contrast, no significant association was found between SINS and functional status as measured by the Barthel Index, Frankel scale, or ECOG performance status at any time point^[22,23,26,27]^ (Table 3).

### 3.5. SINS correlation with HRQoL

Three studies (23%) of those included in this review evaluated the relationship between SINS and HRQoL, using 3 different tools: EORTC QLQ-C30, SF-36, and the EQ-5D. Two studies reported significant associations between SINS and HRQoL measures. Patients with a SINS score >6 one month after radiotherapy experienced significantly greater improvements in QLQ-C30 domains. Higher baseline SINS scores were associated with lower baseline physical functioning on both the SF-36 and EQ-5D, while the pain component of SINS predicted greater improvements in both instruments at 12 weeks posttreatment.^[15,25]^ However, one study reported no significant relationship between SINS category and EQ-5D scores at baseline or posttreatment.^[23]^

### 3.6. Posttreatment SINS correlations stratified by type of intervention

**Figure 2.**
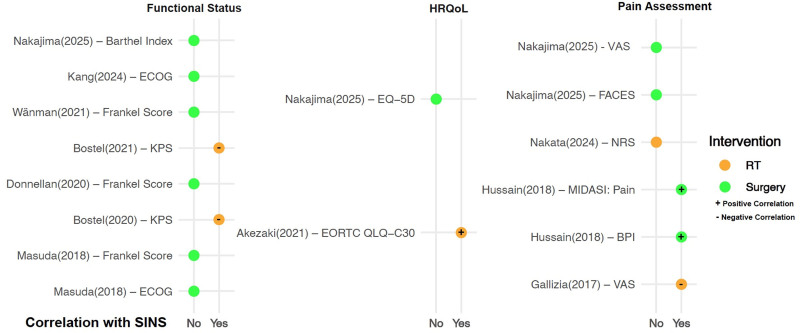
Correlation of the spinal instability neoplastic score (SINS) with patient-centered outcomes across domains of functional status, health-related quality of life (HRQoL), and pain intensity. Green circles represent surgical studies; orange circles represent radiotherapy studies. Plus (+) and minus (–) symbols indicate positive and negative correlations, respectively. BPI = Brief Pain Inventory, ECOG = Eastern Cooperative Oncology Group, EORTC QLQ-C30 = European Organisation for Research and Treatment of Cancer Quality of Life Questionnaire-Core 30, EQ-5D = EuroQol-5D, FACES = FACES pain rating scale, KPS = Karnofsky Performance Status, MDASI = metastatic instability disease assessment of the spine, NRS = numeric rating scale, VAS = visual analog scale.

Seven (54%) studies involved surgery as the main intervention,^[18,20–23,26]^ 5 (38%) involved radiotherapy,^[15–17,19,24]^ and 1 (8%) included both modalities.^[25]^ Among the 7 surgical studies,^[18,20–23,26,27]^ only one reported correlations between postoperative SINS and patient-centered measures, namely MDASI: pain and BPI: worst pain.^[20]^ In contrast, of the 5 radiotherapy-based studies,^[15–17,19,24]^ 4 showed a correlation between posttreatment SINS and patient-centered measures.^[15–17,19]^ One study showed a positive correlation with VAS,^[19]^ another demonstrated a positive association with EORTC QLQ-C30,^[15]^ while the remaining 2 reported negative correlations with functional status measures, specifically KPS and ECOG.^[16,17]^ These findings are summarized in Figure 2. Of note, the study with the hybrid cohort^[25]^ did not assess postoperative correlations so it is not included in this figure.

### 3.7. Risk of bias assessment

Based on the Newcastle-Ottawa Scale, most of the included studies were of moderate to high methodological quality. Specifically, 1 study^[26]^ was rated as high quality (score ≥ 8), while the remaining 12 studies were considered of moderate quality (score 6–7).

## 4. Discussion

SINS was developed to provide a standardized framework for assessing spinal instability, a complication of metastatic spine disease that had been relatively underexplored in the context of surgical decision-making.^[28]^ Ever since, it has been recognized for its utility in predicting the need for surgical fixation and its correlation with vertebral fracture risk.^[13,29]^ It has also demonstrated good interobserver and intraobserver reliability, establishing itself as a highly precise tool that can support consistent clinical decision-making across multidisciplinary teams.^[8]^ Although predicting survival was not one of the intended uses of this scoring system, several studies have investigated its correlation with patient survival.^[23,30]^ To further explore the broader utility of SINS, the present systematic review aims to examine the relationship between SINS and patient-centered measures in individuals with metastatic spine disease.

### 4.1. Pain and SINS

Pain, the only nonradiological component of the SINS scoring system, is the most common complaint among patients with metastatic spine disease.^[31]^ The relationship between baseline pain and SINS was found to be significant in all but 1^[19]^ of the 6 studies^[18–20,23–25]^ evaluating this association. Although this correlation may seem intuitive given that pain itself is a component of SINS, it is crucial to recognize that metastatic spinal disease can produce 2 distinct types of pain. The first type, local tumor-induced pain, arises from periosteal stretching and the release of pro-inflammatory factors, typically presenting as constant pain that is independent of physical activity. In contrast, the second type, mechanical pain, results from spinal instability progression, is positional, and varies with movements.^[32,33]^ SINS places greater emphasis on mechanical pain, assigning it a score of 3, whereas occasional nonmechanical pain is given a lower score of 1.^[4]^ Therefore, a stable lesion can cause more pain than an unstable one if other mechanisms that lead to nociceptor activation become more pronounced. In this context, as noted by Gallizia and colleagues,^[19]^ no significant correlation was identified between preradiotherapy pain intensity and baseline SINS (*P* = .4). We also hypothesize that this observation might be related to higher SINS scores in their cohort due to variables other than pain, namely metastasis location, which was noted at junctional spinal segments in nearly one-third (32.2%) of cases, which would be expected to raise SINS scores without necessarily increasing reported pain intensities. Notably, Gallizia and colleagues also reported that baseline SINS predicted postradiotherapy pain outcomes, with higher initial scores associated with less improvement. In their cohort of 121 patients, they showed that higher baseline SINS scores were linked to less improvement in both pain at rest (*P* = .007) and breakthrough pain (*P* = .047), and that no stable lesions (SINS 0–6) transitioned to the “increased pain” group postradiotherapy.^[19]^

### 4.2. Functional status and SINS

Functional status assessment captures a crucial aspect of metastatic spine disease, as patients often experience a decline in their ability to perform daily activities. These assessments also play a key role in clinical decision-making, as surgeons may choose to forgo surgery in cases where functional status is deemed too poor.^[34,35]^ Although technically a neurological outcome measure, we opted to group Frankel scores under the category of functional status measures, as correlation with ambulation is suggestive of clinically relevant measurement of functional independence.^[21]^

At first glance, the relationship between functional status and SINS may appear straightforward, given that certain components of the SINS, namely spinal alignment, vertebral body collapse, and posterolateral involvement, often reflect more advanced stages of cancer that can also significantly hamper physical functioning. However, the included studies in our systematic review showed inconsistent findings. SINS did not predict postoperative changes in the Frankel index in any of the 3 studies that evaluated this relationship.^[22,26,27]^ Although originally designed for patients with spinal cord injuries, the Frankel index was used by these studies as a surrogate for ambulatory function in metastatic spine disease, with grades D and E generally indicating the ability to ambulate. While these studies provided the same verdict on the correlation between SINS and postoperative Frankel scores, their respective methodological limitations should be noted. Wänman and colleagues^[27]^ and Donnellan and colleagues^[26]^ analyzed data derived from patients in the “potentially unstable” and “unstable” SINS categories, as their cohorts did not include patients with “stable” spines (i.e., SINS < 7). This likely skewed the study populations toward patients with more advanced disease in whom recovery of ambulatory function may be less achievable. This hypothesis is reinforced by the observation of significant improvement in the postoperative Frankel index in the “potentially unstable” group while the “unstable” group did not show a significant improvement.^[27]^ Masuda and colleagues^[22]^ used a binary classification system, grouping patients as “stable” (SINS < 12) or “unstable” (SINS ≥ 12). Although this approach simplified the interpretation, it also eliminated the distinction between the “stable” and “potentially unstable” groups, a clinically significant gap. Prior work has shown that differentiating between these 2 groups can be decisive in surgical decision-making, underlying the clinical importance of this nuance.^[36]^

Regarding KPS and ECOG, while Bostel and colleagues indicated that a KPS score ≥ 70% was significantly associated with a 6-fold higher likelihood of having a stable spine (i.e., SINS < 7) at 6 months postradiotherapy in patients with metastatic head and neck cancers^[16]^ as well as lung and breast cancers,^[17]^ a separate analysis by Kang and colleagues reported no significant correlation between SINS categories and ECOG performance status at baseline or postoperatively.^[21]^ Similarly, Masuda and colleagues observed comparable postoperative improvements in ECOG scores between patients with SINS ≤ 12 and those with SINS > 12, indicating no significant correlation between ECOG status and SINS.^[22]^ We believe the findings reported in both studies by Bostel and colleagues should be interpreted with caution. Both studies used univariate modeling, which is highly susceptible to confounding. One of the most important confounders is overall survival, which was relatively short in their cohorts, 10.8 months in 1 study^[16]^ and 4.8 months in the other.^[17]^ Overall survival plays a key role in this context, as patient survival needs to be long enough to experience potential stabilization-related benefits following radiotherapy. Radiotherapy typically requires roughly 6 months to produce meaningful stabilization.^[37,38]^ However, as the authors reported, only 46 percent of patients were alive at the 6-month follow-up.^[16]^ Patients with higher KPS were more likely to survive longer, making them disproportionately represented in the group with observed stabilization. In contrast, patients with lower KPS may have died before any potential stabilization could occur and only 3 patients in this group achieved stabilization.^[16]^ That said, given the high number of confounding variables, including tumor type, presence of concomitant metastases, radiation dose, and adjunct chemotherapy,^[16,17]^ a multivariate analysis was likely not feasible in their setting, and the reliance on univariate analysis should be viewed in that context.

The Barthel Index, MDASI, and SOSGOQ2.0, were each evaluated in only a single study and demonstrated varying degrees of correlation with SINS. As the first attempt to examine the relationship between SINS-defined spinal instability and patient-reported symptoms, Hussain et al^[20]^ conducted a prospective cohort study using the MDASI, a validated symptom inventory for patients with malignancy. Their findings revealed a significant baseline correlation between SINS and the MDASI domains of walking and activity. However, this association did not persist postoperatively. This may be attributed to the fact that the majority of their cohort (70%) presented with high-grade spinal cord compression, which often improves more slowly, if at all. In contrast, mechanical stabilization achieved through surgery may lead to more immediate relief, rendering the initial association between SINS and MDASI nonsignificant. The physical function domain of the SOSGOQ2.0, a spine-specific patient-reported outcome measure, demonstrated a significant correlation with baseline SINS scores.^[25]^ In contrast, Nakaima and colleagues reported no significant correlation between the Barthel Index and SINS, either at baseline or during the postoperative period. This discrepancy may be explained by the fact that the Barthel Index is a general functional assessment tool and may lack the sensitivity to detect spine-specific impairments.^[39]^

### 4.3. HRQoL *and SINS*

Akezaki et al^[15]^ reported that patients with a baseline SINS score >6 had significantly higher odds of improvement in the global health status domain of the EORTC QLQ-C30 one month after radiotherapy. However, no significant associations were observed between SINS and other EORTC QLQ-C30 domains. The authors classified any patient with a SINS score >6 as “unstable,” effectively combining the “potentially unstable” and “unstable” categories into a single group. Additionally, they extracted data for only 3 domains of the EORTC QLQ-C30, omitting key domains such as social functioning. This limited scope may omit important aspects of this particular HRQoL instrument. The studies by Versteeg and colleagues^[25]^ and Nakajima and colleagues^[23]^ reported conflicting results regarding the relationship between baseline SINS and HRQoL. Versteeg and colleagues^[25]^ found that higher baseline SINS scores were significantly associated with lower physical functioning on the SF-36 and lower EQ-5D scores at baseline. In contrast, Nakajima and colleagues found no correlation between SINS categories (stable, potentially unstable, or unstable) with either baseline or postoperative EQ-5D scores. This inconsistency may be partly explained by the different versions of the EQ-5D used. Versteeg and colleagues utilized the 3-level EQ-5D, which includes fewer response categories and may be more readily affected. Nakajima and colleagues, however, employed the 5-level version (EQ-5D-5L), which offers more nuanced response options but may introduce greater variability, potentially reducing its sensitivity to detect clear correlations with SINS.^[23]^

### 4.4. Stratifying the included studies by primary treatment

The 2 primary treatment modalities for patients with metastatic spinal disease are surgery and RT, though selecting the appropriate approach can be challenging. Traditionally, surgical intervention has been considered for patients with an expected postoperative survival of at least 3 months,^[40]^ as this timeframe is generally required to justify the risks and allow for meaningful recovery following major spinal procedures. In patients with limited life expectancy, RT is often preferred as a less invasive means of symptom relief. Surgery is primarily aimed at decompression and restoration of mechanical stability, typically through spinal fixation techniques along with laminectomy, facetectomy, and, when indicated, circumferential decompression of the spinal cord.^[20]^ In contrast, RT focuses on tumor cytoreduction and re-ossification of lytic lesions.^[41]^ It can be delivered via conventional external beam radiotherapy, typically used for palliative purposes, or stereotactic body radiotherapy, which is a more precise, ablative modality often employed in cases of oligometastatic disease.^[42]^

Stratifying the results of the included studies by treatment modality allows for a more nuanced understanding of how spinal instability correlates with patient-centered outcomes. However, it is important to recognize that the SINS represents only one dimension of clinical decision-making. As emphasized in the Neurologic, Oncologic, Mechanical, and Systemic (NOMS) framework,^[43]^ SINS is integrated alongside other critical factors, such as epidural tumor extension, tumor radiosensitivity, and overall systemic health status, to provide a more comprehensive assessment.

In this context, the contribution of Versteeg et al^[25]^ is particularly valuable, which is the only included study that reports a hybrid cohort treated with both surgery and radiotherapy. Their findings demonstrated that SINS was moderately but significantly associated with baseline pain, physical function, and HRQoL. Interestingly, the majority of studies in this review that employed RT as the primary intervention (Fig. 2) reported posttreatment correlations between SINS and outcomes in all 3 domains. We hypothesize that this pattern may be due to differences in the temporal dynamics of recovery between surgery and RT. Surgical intervention can provide rapid mechanical stabilization, while improvements in patient-centered outcomes may lag. Conversely, RT relies on a slower biological process of re-ossification to achieve structural stability. This gradual improvement, coupled with a generally less aggressive recovery curve, may provide a more synchronized trajectory between changes in SINS and patient-centered outcomes, resulting in more consistent correlations posttreatment.

This study has several limitations, the most prominent being the heterogeneity among the included studies, which precluded a meaningful quantitative synthesis. One major source of heterogeneity lies in the variation of patient-centered measures used across studies. Different tools were employed to assess pain, function, and HRQoL, making direct comparisons difficult. Additionally, clinical heterogeneity among patient populations further complicates interpretation. Patients were at varying stages of disease progression, had metastases from primary tumors with differing radiosensitivities, and may have received a range of treatments beyond surgery and radiotherapy, including systemic therapies and bone-modifying agents.^[20]^ Follow-up durations also varied substantially, influencing the timing and nature of outcome assessments. While this heterogeneity limits standardization, it is somewhat reassuring in that it reflects the real-world complexity of this patient population, as noted by Versteeg et al.^[25]^ Another important limitation concerns the SINS scoring system itself. Despite its clinical utility and incorporation into multidisciplinary frameworks like NOMS,^[43]^ SINS has known limitations. It is primarily designed to assess a single spinal lesion, which complicates its use in patients with multiple metastatic sites, each potentially requiring a separate score.^[20]^ Furthermore, patients with metastatic spine disease often experience a broad spectrum of symptoms not directly attributable to spinal involvement, such as side effects from systemic therapies or symptoms related to metastases in other organs.^[20]^ These factors can confound the interpretation of patient-centered measures, highlighting the importance of using spine-specific symptom inventories, such as the spine tumor module of MDASI, to ensure more accurate and relevant assessments.^[20]^

## 5. Conclusion

This systematic review highlights a meaningful, though nuanced, relationship between the SINS-defined spinal instability and patient-centered measures in individuals with metastatic spine disease. Most notably, consistent preoperative correlations between higher SINS scores and greater pain intensity, reduced functional status, and lower HRQoL all together suggest that SINS, though primarily a radiological tool, carries genuine clinical relevance. These findings support its utility beyond surgical decision-making and its potential value as a proxy for symptom burden and patient experience. However, postoperative correlations between SINS and patient-centered outcomes were less robust, likely due to the heterogeneity of treatment modalities, variable follow-up durations, and the complex, evolving symptom profiles typical in this patient population.

## Author contributions

**Conceptualization:** Hassan Darabi, Harshit Arora, Aysha Alsahlawi, Jared T. Wilcox.

**Data curation:** Hassan Darabi, Harshit Arora.

**Investigation:** Hassan Darabi.

**Methodology:** Harshit Arora, Henry Ruiz-Garcia.

**Supervision:** Jared T. Wilcox, Francis Farhadi.

**Validation:** Henry Ruiz-Garcia.

**Writing – original draft:** Hassan Darabi.

**Writing – review & editing:** Hassan Darabi, Harshit Arora, Aysha Alsahlawi, Francis Farhadi.
